# Open Dialogue compared to treatment as usual for adults experiencing a mental health crisis: Protocol for the ODDESSI multi-site cluster randomised controlled trial

**DOI:** 10.1016/j.cct.2021.106664

**Published:** 2022-02

**Authors:** Stephen Pilling, Katherine Clarke, Georgie Parker, Kirsty James, Sabine Landau, Timothy Weaver, Russell Razzaque, Thomas Craig

**Affiliations:** a Centre for Outcomes Research and Effectiveness, Department of Clinical, Health and Educational Psychology, University College London, 1-19 Torrington Pl, London WC1E 7HB, UK; b Institute of Psychiatry, Psychology and Neuroscience, Kings College London, 16 De Crespigny Park, London SE5 8AB, UK; c Middlesex University, Department of Mental Health & Social Work, Room TG70, Ground Floor Town Hall, School of Health and Education, The Burroughs, London NW4 4BT, UK; d North East London Foundation Trust, Research and Development Department, 1st Floor Maggie Lilley Suite, Goodmayes Hospital, Barley Lane Ilford, IG3 8XJ London, UK

**Keywords:** Open dialogue, Severe mental disorders, Relapse, Crisis care, Social network, Community mental health care

## Abstract

**Background**

‘Open Dialogue’ is a social network model of crisis and continuing mental healthcare which involves elements of service delivery such as immediate response and a style of therapeutic meeting called network meetings. Although there are indications from non-randomised studies that it may help people in their recovery from severe mental health crises and improve long-term outcomes, this has yet to be tested in a randomised controlled trial.

**Methods**

This paper outlines the protocol for a multi-site cluster-randomised control trial assessing the clinical and cost-effectiveness of Open Dialogue compared to treatment as usual (TAU) for individuals presenting in crisis to six mental health services in England. The primary outcome is time to relapse, with secondary outcomes including measures of recovery and service use. Participants will be followed-up for two years, with data collected from electronic medical records and researcher-led interviews. The analysis will compare outcomes between treatment groups as well as investigating potential mediators of effect: shared decision-making and social network quality and size. Carers of a subsample of participants will be asked about their experiences of shared decision-making, carer burden, and satisfaction.

**Discussion**

This trial will provide evidence of whether Open Dialogue services implemented in the English mental health system is an effective alternative to current care and may have important implications for the organization of community mental health services.

**Trial registration**: retrospectively registered (108 participants recruited of 570 target) on 20/12/2019, ISRCTN52653325.

## 1 Introduction

Mental health conditions often involve notable changes in behaviour, thinking and emotion associated with distress, functional impairment and reduced quality of life [4,21,57]. A mental health crisis in this study is defined as a mental health emergency, requiring an urgent response arising from a high risk of harm to self or others or a rapid increase in symptoms of psychosis or severe mood disorder, and which have a significant impact on personal or social functioning.

Those experiencing a mental health ‘crisis’, who are deemed to be at immediate risk to themselves (including suicide) or others or whose symptoms are rapidly worsening often require urgent specialist mental health support [53]. In the English system of state-funded healthcare, there are functionally distinct teams for treating those whose mental health needs are greater than can be met by primary care services [38]. Prompt access to care for those experiencing a crisis may lead to better long-term outcomes and reduced relapse [13,35]. In the English mental health care system this led to the development of Crisis and Home Treatment Teams [31] to provide short-term, intensive support as an alternative to hospital admission, alongside Community Mental Health Teams [17] which could provide long-term care if needed.

However, the need to move between different services during what can be an isolating and disturbing experience for an individual and their family and carers [23,47] has been criticised, with service-users reporting dissatisfaction with the quality of care they have received in the current system [9,26,37]. Given that on-going relationships with professionals has been identified as fundamental to recovery for service users [14,52], it is not surprising that disruptions to continuity of care may result in poorer mental health outcomes [34,52]. Furthermore, there are questions around the effectiveness of these distinct teams and whether they are able to meet their specific functions, with a recent survey finding only 19% of service users reporting a ‘very good’ experience with their community mental health team [10] and mixed evidence on whether crisis teams reduce the need for hospital admissions [28,29].

Open Dialogue is a person-centred, social network model of crisis and continuing mental healthcare where continuity of care is a key organisational principle [48]. It is both a therapeutic practice and a way of organizing a mental health service, and follows seven principles such as ‘immediate help’ and ‘psychological continuity’ [56]. The central means of intervention delivery is through ‘network meetings’: conversations between the service user, those important to them (such as family, formal and informal carers, and friends), and typically two Open Dialogue practitioners who remain with the same network throughout an episode of care [1]. These meetings encourage a shared understanding of the problems leading to a person's contact with the service and shared decision-making about any pharmacological, psychological, or social interventions [56]. It is suggested that this may increase feelings of agency, and the ability to develop and maintain mutual supportive relationships in the longer term [56].

Non-randomised studies have suggested that Open Dialogue may be associated with better mental health outcomes and reduced hospital admissions, ([22,56,50]. However, there is not yet the high-quality evidence for the clinical or cost effectiveness of Open Dialogue to support its widespread implementation [20]. This paper presents the design of and methods for a pragmatic cluster randomised controlled trial, which seeks to provide this evidence for Open Dialogue teams implemented in the English National Health Service (NHS).

## 2 Trial design

This is a multi-site, two-arm, cluster randomised (1:1) controlled trial examining whether Open Dialogue is more clinically and cost effective than treatment as usual (TAU). It forms part of the ODDESSI (Open Dialogue: Development and Evaluation of a Social Network Intervention for Severe Mental Illness) research programme, which also includes development work, a feasibility trial, and a process evaluation.

### 2.1 Objectives

Primary objective: To examine whether Open Dialogue is more effective than TAU at increasing time to relapse after recovery.

Secondary objectives: To examine whether Open Dialogue is more effective than TAU at:

- Reducing time to initial recovery, increasing overall days spent in recovery, and increasing service-user defined recovery
- Reducing service use and societal costs and improving health-related quality of life
- Increasing service-users' satisfaction with care

We will examine whether social network quality, social network size or shared decision-making mediate the impact on the primary outcome. Additionally, we will examine the experience of carer burden, shared decision-making, and satisfaction with care for the family/carers of a subsample of trial participants offered Open Dialogue or TAU.

### 2.2 Study setting and timeline

The trial takes place in six mental health services from inner and outer London, a large university town in the southeast of England and the surrounding suburban and rural communities, and a large costal conurbation in the south-west of England and its surrounding rural communities. The populations served vary considerably in the degree of social deprivation, available community resources and the structure of local mental health services. To commence recruitment, services must meet adequate fidelity to the model of care (see ‘monitoring intervention delivery’).

Participant recruitment commenced in the first site in June 2019, and all other sites were able to open to recruitment by January 2020. The protocol was registered during this set-up period (in December 2019). Recruitment was suspended in March 2020 (to reduce pressure on health services in the initial months of the COVID-19 pandemic) and restarted across the six sites from August 2020 to December 2020. Recruitment will stop in December 2021, the primary outcome will be available from December 2023, and data analysis will commence in early 2024.

### 2.3 Trial clusters and randomisation

Trial clusters are defined in relation to groups of general practices (clinics where family physicians see patients). Inclusion criteria are as follows:

• Each cluster must consist of two to four geographically co-terminous general practices (their catchment areas share a geographical boundary). There must be a clear and pre-existing shared referral pathway to a community mental health service, and a total of 65–75 patients per year referred to mental health crisis services.
• Each general practice within the cluster should refer only within the catchment area of the community mental health service, refer at least 10 people per year to mental health crisis services, and have 2000 or more patients registered with the practice.

Once a mental health service has been recruited, GP practices within its catchment area are assessed and grouped into clusters in line with the cluster eligibility criteria. Relevant details of all the clusters from each catchment area are then sent to the trial statistician who randomly allocates in a 1:1 ratio to Open Dialogue or TAU. Cluster randomisation is stratified by catchment area of the local mental health services and restricted randomisation [11] is used to balance two continuous aggregated cluster-level covariates ‘average GP list size’ and ‘average IMD deprivation rate 2015’ [46] across the two trial arms and the strata. Where there are changes to general practices that form the basis of clusters (for example, two initially separate general practices merging to form a single practice) after cluster allocation, we aim to preserve selection of participants from the original clusters as far as possible.

### 2.4 Participants' eligibility criteria

Participants who present to one of the participating mental health services who meet criteria for the crisis and continuing care pathway, and who are registered with a GP within a trial cluster are provided with Open Dialogue or TAU depending on their cluster. They may present by any route permitted within the service's local policies and procedures (for example, referral by a GP, an Emergency Department or other specialist service, or admission into an inpatient ward). There are further eligibility criteria for research participation:

• Evidence of a mental health crisis indicated by (a) a rating of A-C on the UK Mental Health Triage Scale [53], which indicates either an emergency, very urgent or urgent response is needed based on a high risk of harm self or others and/or rapidly increasing symptoms of psychosis or severe mood disorder**,** (b) an admission to an inpatient ward or (c) being accepted into the care of a mental health crisis service
• 18 years of age or more
• Sufficient English language abilities to participate in the research

Exclusion criteria are as follows: a diagnosis of dementia or an acquired cognitive impairment, a primary diagnosis of a learning disability or substance misuse, having no fixed abode, being under the care of forensic mental health services, and having previously received Open Dialogue.

Recruitment to the study was also not possible for those who are already participating in another research project that may impact their care in this trial, or if recruitment to the study would pose a risk of harm to the participant, study clinicians, or researchers.

### 2.5 Outcomes

The primary outcome is time, in days, to ‘relapse’ following recovery. Recovery is defined as the absence of significant symptoms and presence of adequate social functioning; and relapse as the return of significant symptoms and deterioration of social functioning [7]. A summary of each participant's social circumstances, mental health, and type of response from health care providers for the two-year follow-up period is created primarily from their electronic medical records and research contacts. This summary is reviewed by a panel of trained raters, blind to treatment allocation, who use it to estimate the presence and date of any recovery or relapse using an established method [7]. Any information that might unblind the panel is redacted, and the panel's estimation of allocation is monitored.

For a description of secondary outcomes, measures, potential mediators, and family/carer measures see Table 1 below.

**Table 1:** Details of secondary outcomes, potential mediators, and family/carer measures.

	Outcome	Measure	Description of measure
Secondary outcomes	Recovery	Time in days to initial recovery, and the total number of days spent in recovery [7]	An expert panel estimate based on 24 monthly summaries of a person's social circumstances, mental health, and type of response from healthcare practitioners. See assessment method described for the primary outcome measure.
	Service-user defined recovery	Questionnaire on the Process of Recovery (QPR) [40]	A 15-item self-report measure developed to assess intrapersonal and interpersonal functioning, measured at 3, 6, 12, and 24 month time points (test-retest reliability *r* = 0.769 [40]).
	Health-related quality of life	EQ-5D-5L [25]	A self-report measure yielding a 5-digit number describing self-rated health state measured, during the first research interview, and at 3, 6, 12, and 24 months post initial crisis presentation **(**intraclass correlation coefficient = 0.75 [30]).
	Service use and societal costs	Client Service Receipt Inventory (CSRI) [8] Plus the number of and length in days of psychiatric admissions, and number of accepted referrals to crisis services in the 24 months after the initial crisis	A tool used to collect information on service utilisation, accommodation, income, and other costs measured at 3, 12, and 24 months post the initial crisis presentation.
	Service user satisfaction	Client Satisfaction Questionnaire (CSQ-8) [6]	A self-report measure of service user overall satisfaction with care measured at 3, 6, and 24 months post initial crisis presentation (alpha coefficient, 0.83 to 0.94 [2]).
Potential mediators	Social network quality and size	Social Provisions Scale (SPS) [15]	A 24-item self-report measure that examines the degree to which respondent’s social relationships provide the dimensions of social support using a model of social provisions [60] measured during the first research interview, and at 3, 6, 12, and 24 months post initial crisis presentation. Scores range between 24 and 96 with a higher score indicating a greater degree of perceived support indicative of higher quality social networks (alpha coefficient, 0.89 [12]).
		Lubben Social Network Scale (LSNS-6) [33]	A 6-item self-report scale that assesses the size, closeness, and frequency of contacts of a service user's social network (family and friends), providing a total score between 0 and 30 during the first research interview, and at 3, 6, 12, and 24 months post initial crisis presentation. (alpha coefficient, 0.78 [12]).
	Shared decision making	The Modified Dyadic OPTION scale [36]	Assesses the degree to which clinicians involve patients in shared decision making through observer rating of clinician behaviour, measured at 3, 6, and 24 months post initial crisis presentation (alpha coefficient 0.9 [41]).
Family/carer outcomes	Carer satisfaction	Client Satisfaction Questionnaire (CSQ-8) [6]	A self-report measure of carer overall satisfaction with care measured at 3, 6, and 24 months.
	Carer burden	The Burden Assessment Scale [49]	A 19-item self-report measure completed by the primary caregiver to assess perceived objective and subjective consequences of providing care to a service user, measured at 3, 6, and 24 months, (alpha coefficient, 0.92 [27]).
	Shared decision making	The Modified Dyadic OPTION scale [36]	Assesses the degree to which clinicians involve carers in shared decision making through observer rating of clinician behaviour, measured at 3, 6, and 24 months.

We also collect diagnoses and service use in the 6 months before the index crisis (from medical records), and baseline demographic information and the Brief Psychiatric Rating Scale (BPRS), which assesses symptoms of severe mental illness by interview and observation. Scores range from 18 to 126 [45] (intraclass correlation = 0.78, [5]). Intervention receipt is logged for each participant throughout trial participation.

#### 2.5.1 Sample size

Thirty clusters, with 19 participants each, provide an initial sample size of 570 participants, with a projected 540 participants being observed during the follow-up period. Following Freedman's [19] formula, a total of 157 participants was calculated as being needed to relapse during the 24-month follow-up period to detect an HR = 0.589 or more, with 90% power using a log-rank test at the 5% significance level. Following a method by Rutterford, Copas, and Eldridge [51], this number was inflated by our design effect (design effect = 1 + (obs. Per team cluster - 1) ICC = 1 + 17 × 0.04 = 1.68; based on a typical intra-cluster correlation in cluster randomised trials of ICC = 0.04; [18] to calculate the total number of relapses needed to adjust for clustering. This gives a requirement of 264 relapses during the 24-month follow-up period. Assuming an average relapse rate of 49%, this requires an effective sample size of 264/0.49 = 538 participants.

#### 2.5.2 Blinding

The senior statistician, trial Chief Investigator (CI), and those assessing the primary outcome measure will remain blind to cluster allocation throughout the trial. All trial statisticians are blind to allocation during the randomisation process and prior to the approval of the Statistical Analysis Plan (SAP). Researchers collecting outcome data from participants and working with clinical teams are not blind to cluster allocation.

### 2.6 Statistical analysis

The primary outcome, time to relapse from recovery, will be analysed using Cox regression, where time to relapse will be the dependent variable, with fixed explanatory variables of trial arm, and stratification factor (catchment area of the mental health service), and cluster-level balancing variables of general practice list size, and Index of Multiple Deprivation. The model will also contain a random intercept that varies at the level of the catchment area to account for general practice cluster effects.

This modelling will supply an estimate of the hazard ratio of relapse comparing Open Dialogue with TAU and an associated 95% confidence interval. In the (unlikely) event that covariates are missing or that aspects of the Open Dialogue intervention (e.g. number of contacts between participants and teams) drive censoring times, we will employ multiple imputation for survival outcomes as described in White and Royston [61].

Continuous secondary outcomes measured at several time points will be analysed using linear mixed models. In these models the available post-allocation measures of the secondary outcome variables form the dependent variable, with trial arm, time, trial arm x time interactions terms, stratification, and balancing variables as explanatory variables. To account for the correlation between repeated measures taken on the same subject, subject-varying random effects will be included in the model. To account for remaining correlation between outcomes from patients from the same cluster, a further cluster-varying random intercept will be included in the model.

Distributional assumption will be checked and continuous outcomes arising from skewed distributions suitably transformed before analysis.

#### 2.6.1 Methods for additional analyses

Mediation modelling will be performed to investigate the hypothesis that improvements in times to relapse under Open Dialogue are brought about by enhanced quality/size of the social network or by higher levels of shared decision-making. Specifically, we will estimate the direct (non-mediated by networking) and indirect (mediated by networking) effects of Open Dialogue on time to relapse. For this purpose, social network quality, social network size, and shared decision-making at 6 months will be assessed as potential mediators. Confounders of the relationship between network quality and relapse (e.g. social network score or symptom severity at first research interview) will be measured and included in the mediation modelling. A complier average causal effect (CACE) analysis will also be undertaken to estimate the effect of actually receiving the intervention using instrumental variables regression.

#### 2.6.2 Statistical methods to handle missing data

All formal statistical analyses will follow the intention-to-treat principle. Estimates derived by fitting linear mixed models are valid in the presence of missing outcome values provided the missing data generating mechanism is missing at random (MAR). This particular MAR assumption allows all variables included in the analysis model to drive missingness. Multiple imputation will be employed should covariates be missing, or aspects of the interventions drive absence of outcomes. Details of any additional analyses will be described in the SAP.

## 3 Intervention delivery

Open Dialogue teams offer care from initial presentation in crisis through to discharge from the service, typically through ‘network meetings’ which include the service user, people they identify as important to them such as family members, and two or more Open Dialogue Practitioners. The agenda is set by the network, and there is a strong emphasis on service-user participation in any treatment decisions. Wherever possible, the same practitioners remain with the network throughout. Teams are multidisciplinary, typically including nurses, social workers, peer practitioners, clinical psychologists, occupational therapists, and psychiatrists, all of whom have undertaken an Open Dialogue training programme. Services are underpinned by the seven key principles of Open Dialogue: (1) Immediate Help; (2) A Social Network Perspective; (3) Flexibility and Mobility; (4) Responsibility; (5) Psychological Continuity; (6) Tolerance of Uncertainty; and (7) Dialogue and Polyphony [56]. They are ‘needs-adapted’ and network meetings take place as little or often as required by the network, perhaps occurring twice weekly in the time immediately after referral, moving to weekly or fortnightly as treatment progresses, whilst also working flexibly in collaboration with other services [48]. There is an ‘open discharge policy’, with service users able to ask for further network meetings at any point after discharge from the service, ideally with the same practitioners.

TAU is current routine crisis care and continuing community care, as delivered by English mental health services. This is based on a model which comprises a number of multi-disciplinary teams, the provision of care coordination, and a range of community and in-patient interventions and support.

 Participants in both trial arms may be offered any intervention they would ordinarily have access to individual psychological and pharmacological interventions, social, and housing support. The duration of contact is determined by each team, based on clinical need. Those with complex problems may receive continuing care for the duration of the study. Open Dialogue is not offered to those in the TAU arm. The provision of mental health services after the follow-up period has ended will be the responsibility of the participants' current mental healthcare provider.

All teams in the trial were required to deliver care adequately (according to adherence and fidelity criteria detailed below), and to produce a site-specific operational policy which described care pathways and met trial requirements for Open Dialogue services prior to recruitment of trial participants. All Open Dialogue trained staff are offered annual refresher training.

### 3.1 Monitoring intervention delivery

The fidelity of service delivery in both the Open Dialogue and TAU teams is assessed to ensure that that the core structural components of services are delivered as intended. The ODDESSI fidelity measure [3] draws on an existing measure [44], and is based on interviews with service staff and review of documentation. Four key aspects of service functioning are rated: (1) Team structure and culture, (2) Access and engagement, (3) Delivery of care, (4) External support. To meet fidelity services must score at least 2.4 of 4 on each of the above.

The extent the core therapeutic components of Open Dialogue are delivered within a network meeting is measured using the Open Dialogue Adherence scale [32] which was developed from (and closely aligned with) a previous adherence measure [44]. Audio recordings are assessed by trained raters and scored according to the key elements of dialogic practice [32,43] and then rated as ‘acceptable’ or ‘unacceptable’. In ‘acceptable’ tapes at least two thirds of statements used by practitioners are dialogic (rather than monologic) and less than two statements are patronizing or disrespectful, and at least 10 out of 12 of the items based on the key elements of dialogic practice score ‘acceptable’.

All Open Dialogue and TAU teams are monitored prior to participant recruitment and at 6-monthly intervals throughout intervention delivery. Where fidelity or adherence is identified as falling below the pre-determined levels, remedial action is taken, and recruitment may be stopped locally in that cluster if the problem persists despite remedial action. Actual intervention or ‘dose’ received: the number, date, and mode of delivery of clinical meetings, is also recorded at a participant level.

## 4 Research processes

### 4.1 Participant recruitment and retention

If a person presents to services and appears eligible, a local staff member asks if a researcher can make contact. Those who agree are contacted by a researcher with further information, and after time to consider (usually 48 h or as long as they need), they are asked for informed consent. If they lack capacity, consent via a personal (relative or friend) or nominated consultee (a local senior clinical professional consultee with no other involvement with the trial) may be sought [58], and reviewed at the point they regain capacity. Separate informed consent is sought from the family and carers of a purposive sub-sample of trial participants for questionnaires, and from all who attend a network meeting to audio-record that meeting.

Participants are also given a £15 shopping voucher for each interview and are reimbursed for any additional expenses (e.g. travel). For those participants who choose to withdraw from further participation, agreement is sought for access to their electronic medical records for the remainder of the study to allow for the primary clinical outcome to be collected.

Research staff are located within NHS sites, closely engage with clinical teams, and receive regular training, support, and supervision to effectively engage participants. Regular meetings with site-based Principal Investigators (PIs) and other senior clinical and management staff, and input from the Lived Experience Advisory Panel (LEAP) address any issues with participant recruitment and retention.

### 4.2 Data collection

Primary data are collected during research interviews and are extracted from electronic medical records (see Table 2 for a full schedule of data collection).

**Table 2:** Data collection time-points.

Data source	Data collected	True baseline (crisis)	First research interview	3 months	6 months	12 months	24 months
RCT participants	Sociodemographic information		X				
	Brief Psychiatric Rating Scale (BPRS)		X				
	Health related quality of life (EQ-5D-5L)		X	X	X	X	X
	Client Services Receipt Inventory (CSRI)			X		X	X
	Social Provisions Scale (SPS)		X	X	X	X	X
	Lubben Social Network Scale (LSNS-6)		X	X	X	X	X
	Questionnaire about the Process of Recovery (QPR)			X	X	X	X
	Client Satisfaction Questionnaire (CSQ)			X		X	X
	Modified Dyadic OPTION scale			X	X		X
Review of records	• Diagnoses (if any) • Drug/alcohol misuse • Contact with secondary mental health services and crisis referrals • Number of hospital visits • Inpatient stays • Other long-term health problems	X (Extracted for the 6 months prior to baseline)					
	Number of clinical meetings	Ongoing throughout trial participation					
	Relapse and recovery summary	Primary outcome assessed at 24 months					
	Hospitalisation	For the 6 months prior to baseline, and at 12 and 24 months					
	Crisis referral	For the 6 months prior to baseline, and at 12 and 24 months					

#### 4.2.1 Baseline and follow-up research interviews

**Fig. 1:**
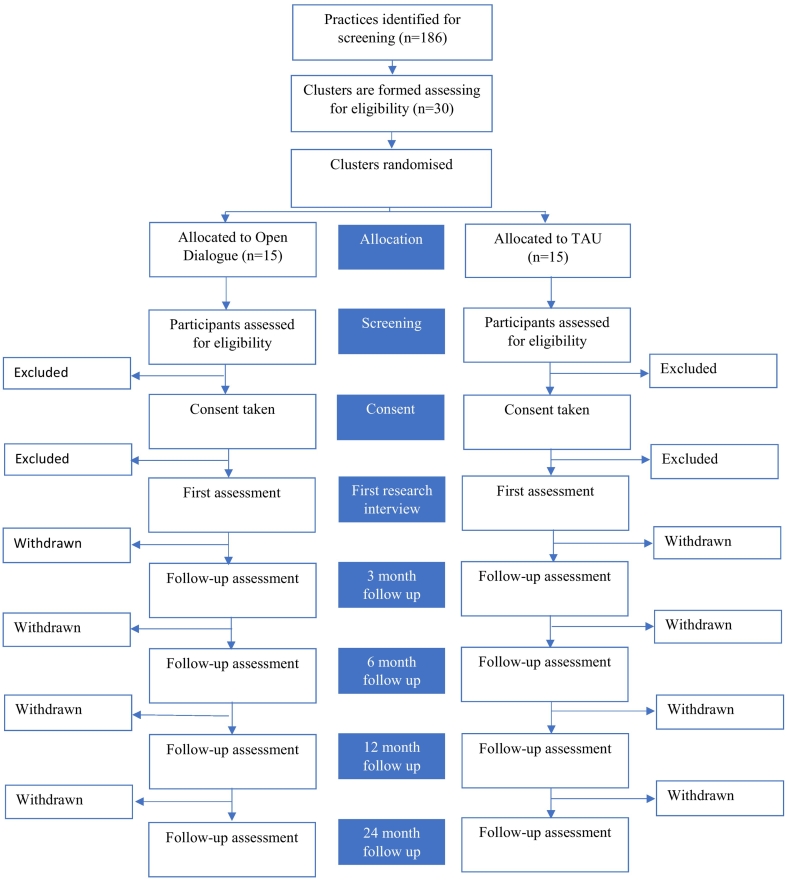
Study design.

The baseline timepoint is the date of the crisis referral. This referral takes place after cluster randomisation, but before a potential participant has been assessed as meeting eligibility criteria prior to consent. The first research interview is conducted as close as possible to the date of the crisis referral (baseline) after consent is provided. Research interviews either occur in person (i.e. at the research site or at participant's homes) or over the telephone (the primary method of data collection during the COVID-19 pandemic). Follow-up interviews are undertaken at 3, 6, 12, and 24 months from baseline (see Fig. 1).

#### 4.2.2 Electronic medical records

After consent, baseline data are extracted from medical records for the date of the crisis referral and for the 6-months prior, and further service use data collected throughout the follow-up period. The monthly summary to be assessed for the primary outcome is created primarily from electronic medical records after completion of trial participation.

#### 4.2.3 Family and carer interviews

Research interviews asking about shared decision-making, carer burden, and satisfaction also take place with a family member or carer of a sub-sample of participants (20%) at 3, 6, and 24 months from the date of the main trial participant's baseline.

### 4.3 Data management

Trial participants' and carer's interview responses are collected on paper Case Report Forms (CRFs) and stored securely in locked filing cabinets, or electronic CRFs saved on secure shared drives on password protected computers within each trial site. Participants are identified only by a study ID on all CRFs (so they are pseudonymised). Files which include participants' names or contact details (consent forms and a trial management log) are stored in a separate physical or digital space. Data are only transferred where needed and each researcher is responsible for following their institution's protocols for safe data transfer. Only the CI and the named research team have access to personal data.

Data for fidelity and adherence ratings are stored electronically in a password protected file on a password protected computer, separate from any personally identifiable information. Recordings are uploaded to the Data Safe Haven at University College London (UCL).

Pseudonymised trial participant data are entered into a database via a web-based data entry system (InferMed MACRO) with access restricted to specific researchers or regulatory authorities, and for a specified purpose. The system is compliant with Good Clinical Practice [24] and has a full audit trail. The research team ensure data quality through regular reviews of the data and will carry out final data quality checks and cleaning before the database is locked prior to data analysis. The full details of the planned statistical analyses will be outlined in the SAP, which will be signed off before database lock.

Participant data will be stored securely for 10 years following completion of data collection. The trial is registered in accordance with the General Data Protection Regulation [16].

## 5 Trial management, oversight, and monitoring

The trial is managed by the Chief Investigator (SP), Principal Investigators in each study site, and a Trial Management Group consisting of co-investigators, trial statisticians and the trial manager. Oversight is provided by a Lived Experience Advisory Panel (LEAP), an independent Programme Steering Committee (PSC), and an independent Data Monitoring Committee (DMC). All changes to trial protocol are reviewed and agreed by the PSC, and DMC if relevant, prior to implementation. Any protocol amendments are submitted to the Ethics Committee for review and if approved, circulated to trial sites and research staff.

The trial sponsor (North East London NHS Foundation Trust) also provides oversight, cover for any harm which may arise from trial participation, and audits the trial.

The reporting of serious adverse events is carried out in accordance to the UK policy framework for health and social care research [24]. Serious adverse events (defined in this trial as an untoward medical occurrence which is life threatening, requires hospitalisation, causes a persistent or significant disability, congenital abnormality, or birth defect or which results in death) will be described from a participant's baseline crisis, following their consent to participate in the study: either on review of patient notes, or as disclosed to researchers.

### 5.1 Changes to protocol

Our initial sample size was 644 participants: 28 clusters each with 23 participants. We subsequently exceeded our cluster recruitment target by 4 clusters, but 2 clusters (the 7th mental health service) withdrew prior to participant recruitment due to the COVID-19 pandemic. Following the pause to recruitment due to the pandemic in 2020 we reviewed the original sample size calculation and recalculated based on 30 clusters and a more accurate estimate of attrition (5% attrition as opposed to the 15% originally used). All other parameters in the sample size calculation remained the same.

The onset of the pandemic meant a rapid move to remote delivery of Open Dialogue and TAU. To assist with this, a Remote Working Guidance Group consisting of national and international experts in Open Dialogue, trial clinicians, services users, and carers collaborated on the development of a Remote Working Protocol [42] which was implemented alongside each site's local remote working protocols. The criteria for adherence and fidelity (detailed above) are equally applied to services before and after the onset of the pandemic.

Research procedures (such as baseline and follow-up participant interviews, attending meetings with the clinical teams, etc.) moved to primarily remote delivery. Additional data are collected on the mode of delivery of clinical meetings (i.e., face-to-face, telephone call, video conference, or a combination) to support an exploration of the impact this has on outcomes.

## 6 Discussion

This paper presents details of the trial design, intervention delivery, and research processes of the first large-scale multi-site cluster randomised controlled trial of Open Dialogue, a person-centred, social network model of crisis and continuing mental healthcare. In this trial, adults presenting in crisis to mental health services in six areas of England are offered either Open Dialogue, or treatment as usual, and their mental health and service use monitored over the subsequent two years.

The trial seeks to assess whether Open Dialogue is more effective in helping individuals with severe mental illness to reach a state of recovery which is robust against relapses than Treatment as Usual. The trial participants are identified on the basis of experiencing a mental health crisis and the need for specialist secondary mental health care, rather than a specific diagnosis. This is not typical of other Open Dialogue and related research (e.g. [1,22,[54], [55], [56]]), but increases the generalisability of the results and the potential that Open Dialogue could provide an alternative to the functional team based model of mental healthcare [17]. In order to achieve this, exclusion criteria are kept to an absolute minimum. The inclusion of measures of cost, and a health economic analysis, will mean the economic viability of Open Dialogue as an alternative to usual care may also be established.

Monitoring of the delivery of Open Dialogue is an important part of this trial's design. The teams were established and deliver care within existing NHS service structures. For this reason the teams vary somewhat in their structure and relationship to existing services and are governed by different Trusts with different local policies and procedures, and varying care pathways. Adaptations to the model have been needed and were set out in operational requirements of an Open Dialogue Service within the NHS, and in particular to ensure that the intervention would be appropriate for those with depression, anxiety, eating disorders and personality disorders, as well as for those with psychotic disorders where much of the early work on Open Dialogue took place [48,56]. Maintaining a high-quality Open Dialogue service in this context can be challenging, particularly where structural changes impact on, for example, the re-organization of in-patient services and the development of a single points of entry for crisis referrals.

The secondary outcomes will allow for an exploration of whether improvements in social networks or involvement in decision making about mental health treatment decisions mediates any treatment effect. It has been suggested that Open Dialogue may improve outcomes through increasing a sense of agency and the ability to develop and maintain mutual supportive relationships [56]. Shared decision-making and social network size and quality will be investigated as potential mediators of treatment effect.

We will publish the results of the trial in a peer-reviewed open access journal article (expected to be early 2024). Further dissemination of findings will include journal articles, conference presentations, patient and public involvement events, and a plain language summary of findings online [59]. If Open Dialogue is found to be more clinically and cost-effective in preventing relapse and aiding recovery in this population, this will have important implications for the organization and delivery of mental health services in the UK and internationally.

## Authors' contributions

Stephen Pilling: Conceptualization, Methodology, Writing - Original Draft, Writing - Review & Editing, Supervision, Funding acquisition. Katherine Clarke: Writing - Original Draft, Writing- Review & Editing, Project administration. Georgie Parker: Writing - Original Draft, Writing- Review & Editing, Project administration. Kirsty James: Formal analysis, Writing - Review & Editing, Methodology. Sabine Landau: Formal analysis, Writing - Review & Editing, Supervision, Methodology, Funding acquisition. Timothy Weaver: Writing - Review & Editing, Supervision, Funding acquisition. Russell Razzaque: Conceptualization, Methodology, Writing - Review & Editing, Supervision, Project administration, Funding Acquisition. Tom Craig: Writing - Review & Editing, Supervision, Funding acquisition.

## Funding

Funding for this research programme is provided by the 10.13039/501100000272National Institute for Health Research (NIHR) PGfAR Project Number: RP-PG-0615-2002. SL and KJ's contributions represent independent research part funded by the NIHR Biomedical Research Centre (South London and Maudsley NHS Foundation Trust and 10.13039/501100000764King's College London) and the NIHR Applied Research Collaboration South London (10.13039/100010872King's College Hospital NHS Foundation Trust). SP is also supported by the 10.13039/501100008721UCLH Biomedical Research Centre.

## Availability of data and material

The researchers will be following the actions outlined by NIHR in regard to sharing research data [39]. The datasets generated and analysed and the corresponding statistical code will be available in anonymised form from the research team on reasonable request, subject to review, following the publication of trial results.

## Ethics approval and consent to participate

This trial has received ethical approval from Wales 5- Research Ethics Committee and is being conducted in accordance with the Declaration of Helsinki. All participants provide informed consent and sign a consent form as part of the first research interview, which specifies that participant details will be made anonymous.

## Consent for publication

Not applicable.

## Declaration of Competing Interest

The authors declare that they have no known competing financial interests or personal relationships that could have appeared to influence the work reported in this paper.
